# Z-Guggulsterone Induces Apoptosis in Gastric Cancer Cells through the Intrinsic Mitochondria-Dependent Pathway

**DOI:** 10.1155/2021/3152304

**Published:** 2021-01-04

**Authors:** Ruxi Lv, Min Zhu, Kun Chen, Haitao Xie, Hongxia Bai, Qingfa Chen

**Affiliations:** ^1^Department of Gastroenterology, Liaocheng People's Hospital, Liaocheng 252000, China; ^2^Clinical Laboratory, Liaocheng People's Hospital, Liaocheng 252000, China; ^3^College of Medicine, Liaocheng University, Liaocheng 252000, China; ^4^Centre for Research, Xiankangda Bio-Tech Corporation, Dongguan 523000, China; ^5^The Institute for Tissue Engineering and Regenerative Medicine, The Liaocheng University/Liaocheng People's Hospital, Liaocheng 252000, China

## Abstract

**Background:**

To study the effects of z-guggulsterone on gastric cancer cell apoptosis and the mechanism related.

**Materials and Methods:**

Human gastric tumor SGC-7901 cells and GES-1 normal epithelial cells were treated with z-guggulsterone (0–75 *μ*M) for 24 h. MTT assay was applied to evaluate cell proliferation. Flow cytometry and Hoechst staining were used to assess cell apoptosis. Western blotting was applied to evaluate FXR, small heterodimer partner (SHP), Bcl-2, and Bax protein expression. ELISA was applied to gain the levels of active caspase-3 and the contents of TNF-*α*, TGF-*β*1, and VEGF.

**Results:**

The expression levels of FXR and SHP were higher in tumor cells than in normal epithelial cells. Inhibition of FXR signaling with z-guggulsterone dose-dependently inhibited SGC-7901 cell proliferation and promoted SGC-7901 cell apoptosis. Bcl-2 protein expression was significantly decreased, and active caspase-3 and Bax protein expression was increased in SGC-7901 cells incubated with z-guggulsterone. The content of TNF-*α* was significantly increased, and the contents of VEGF and TGF-*β*1 were decreased in SGC-7901 cells incubated with z-guggulsterone.

**Conclusions:**

Inhibition of FXR signaling with z-guggulsterone induced anticancer effects in SGC-7901 cells by decreasing cell proliferation and promoting apoptosis. Z-guggulsterone induced cell apoptosis through the mitochondria-dependent pathway.

## 1. Introduction

As a deadly malignancy and the main cause of cancer-related deaths, gastric cancer is becoming a severe worldwide health problem. Until now, gastric cancer still has a poor prognosis in spite of improvements in diagnostic tools and therapy strategies [1]. In China, gastric cancer-related deaths are the highest in the world, whatever men or women [2].

Chemotherapy following surgical excision is the most popular method for gastric cancer therapy, but chemotherapy options are limited due to the fact that these treatments kill not only cancer cells but also normal cells by inducing toxicity and apoptosis [3]. Therefore, many researchers around the world have carried out studies to develop safe reagents that can target cancer cells and induce little damage to normal cells. Naturally occurring compounds have the potential to treat cancer patients [3]. The plant steroid z-guggulsterone, obtained from *Commiphora mukul*, has been used as a farnesoid X receptor (FXR) antagonist to treat obesity, lipid metabolism dysfunction, hypothyroidism, inflammation, and arthritis [4]. Z-guggulsterone induces apoptosis in various cancer cell types such as prostate cancer, acute myelocytic leukemia, colon cancer, liver cancer, glioma, pancreatic cancer, esophageal cancer, and gastric cancer [5–13]. While several studies have demonstrated the antitumor activities of z-guggulsterone, it remains unknown whether z-guggulsterone modulates intrinsic mitochondrial apoptosis in gastric tumor cells.

## 2. Materials and Methods

### 2.1. Cell Culture

Human gastric normal cell line GES-1 and human gastric cancer cell lines SGC-7901 were provided by the Cell Bank of Type Culture Collection (CTCC) of Chinese Academy of Sciences (Beijing, China). These cells were cultured in Dulbecco's Modified Eagle Medium (Gibco, USA) at 37°C and 5% CO_2_ in a humidified incubator.

### 2.2. 3-(4,5-Dimethylthiazol-2-yl)-2,5-diphenyltetrazolium Bromide (MTT) Assay

MTT assay was performed to evaluate the cell viability after the treatment of z-guggulsterone. Cells were plated in a 96-well plate (1 × 10^5^ cells/well) and incubated with 0, 15, 50, and 75 *µ*M z-guggulsterone for 24 h. The results were determined at 490 nm under an ELISA microplate reader (iMark™; Bio-Rad Laboratories, USA).

### 2.3. Determination of Cell Death by Annexin V/Propidium Iodide (PI) Staining and Hoechst 33258 Staining

Gastric cancer cells were plated in 6-well plates (2 × 10^5^ cells/well) and incubated with 0, 50, or 75 *µ*M z-guggulsterone for 24 h. Then, cells were subjected to Annexin V/PI staining (BD Biosciences, USA) and analyzed by using FACS Calibur flow cytometer (BD Biosciences, USA).

### 2.4. Western Blot Analysis

Western blotting was used to detect protein expression as previously described [14]. In brief, cells were collected in lysis A buffer containing phosphatase and protease inhibitors (Beyotime, China). A total of 30 *μ*g of protein was separated by SDS-PAGE, electrotransferred to PVDF membranes, and kept in incubation with primary antibodies overnight at 4°C. The list of primary antibodies was as follows: mouse anti-FXR (#sc-25309, 1 : 750; Santa Cruz), rabbit anti-SHP (#sc-2305, 1 : 750; Santa Cruz), rabbit anti-Bcl-2 (#ab32124, 1 : 500; Abcam), anti-Bax (#ab182733, 1 : 500; Abcam, USA), and mouse anti-*β*-actin (#A1978, 1 : 100; Sigma-Aldrich, USA). Bands in the membranes were determined with enhanced chemiluminescence reagent (Thermo Fisher, USA).

### 2.5. Caspase-3 Activity Assay

Gastric cancer cells were plated in 6-well plates (2 × 10^5^ cells/well) and incubated with 0, 15, 50, or 75 *µ*M z-guggulsterone for 24 h. Enzyme-linked immunosorbent assay (ELISA) was used to determine the level of active caspase-3 following the manufacturer's instructions.

### 2.6. Determination of Cytokines

Gastric cancer cells were plated in 6-well plates (2 × 10^5^ cells/well) and incubated with 0, 15, 50, or 75 *µ*M z-guggulsterone for 24 h. ELISAs were used to determine the contents of VEGF, TNF-*α*, and TGF-*β*1 in gastric cancer cells following the manufacturer's instructions.

### 2.7. Statistical Analysis

For statistical analysis, one-way ANOVA followed by Tukey's test and unpaired Student's *t*-test were performed in GraphPad Prism software. The results are shown as means ± standard deviation (SD). A *p* value of no more than 0.05 was regarded as statistically significant.

## 3. Results

### 3.1. Relative FXR and Small Heterodimer Partner (SHP) Expression in Gastric Cancer Cells

**Figure 1:**
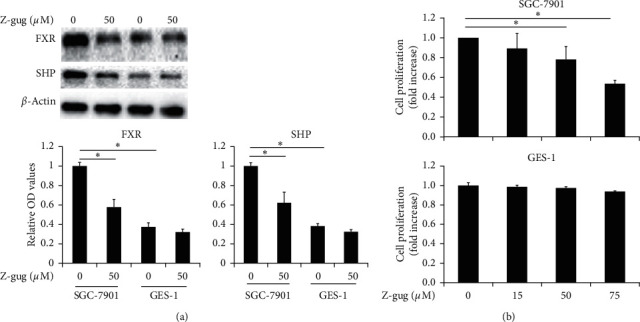
Z-guggulsterone, an FXR antagonist, exhibited cytotoxicity in SGC-7901 cells. (a) Cells were incubated with 0 and 50 *µ*M z-guggulsterone. FXR and SHP protein levels were assessed by western blotting. Quantification of protein bands are shown in the bottom panels. *β*-Actin was used as a control. Data are expressed as means ± SD. ^*∗*^*p* < 0.05 vs. untreated cells. (b) Cells were incubated with 0, 15, 50, and 75 *μ*M z-guggulsterone, and cell survival was investigated using MTT assay. Data are expressed as means ± SD. ^*∗*^*p* < 0.05 vs. untreated cells. Z-gug, z-guggulsterone.

We analyzed FXR expression level in gastric cancer cells. The expression of FXR was significantly higher in SGC-7901 cells comparing with that in normal gastric GES-1 cells (*p* < 0.05) . Z-guggulsterone, an FXR antagonist, significantly inhibited the expression of FXR in SGC-7901 cells (Figure 1(a)). Nuclear receptor SHP is a classic downstream molecular of FXR during metabolism and cancer progression. We then analyzed the expression of SHP in gastric cancer cells. The result showed a higher expression of SHP in SGC-7901 cells when compared to that in normal gastric cells (*p* < 0.05), and z-guggulsterone significantly suppressed the expression of SHP in SGC-7901 cells (Figure 1(a)). This difference demonstrates a higher FXR signaling in gastric cancer cells.

### 3.2. Z-Guggulsterone Exhibited Potent Antitumor Activity in SGC-7901 Cells

To investigate the possible roles of FXR in gastric cancer, the viability of SGC-7901 cells was analyzed following treatment with z-guggulsterone, an FXR antagonist. The results indicated that z-guggulsterone led to dose-dependent cytotoxicity in SGC7901 cells (Figure 1(b)). The survival rate of SGC-7901 cells was approximately 50%, while GES-1 cells were only minimally affected by incubation with 75 *µ*M z-guggulsterone (Figure 1(b)). This indicated that z-guggulsterone targets tumor cells with less cytotoxicity to gastric epithelial cells, suggesting z-guggulsterone may be a safe and useful anticancer compound.

### 3.3. Z-Guggulsterone Induces Apoptosis in SGC-7901 Cells

**Figure 2:**
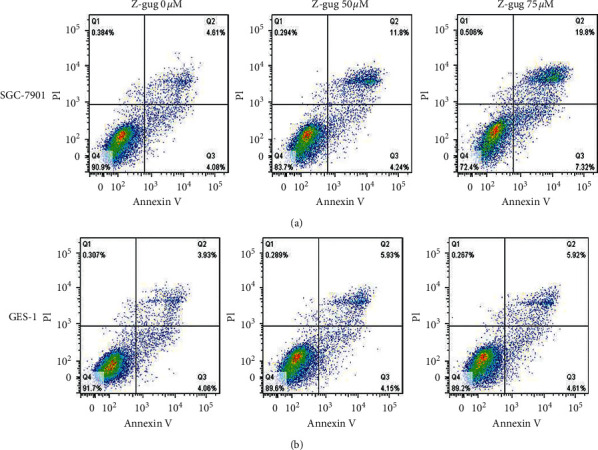
Z-guggulsterone triggered apoptosis in SGC-7901 cells. SGC-7901 (a) and GES-1 (b) cells were incubated with 0, 50, and 75 *μ*M z-guggulsterone for 24 h, and cell apoptosis was investigated by Annexin V/PI staining. Z-gug, z-guggulsterone.

To understand whether z-guggulsterone induces gastric cancer cell apoptosis, we performed Annexin V/PI staining following treatment with z-guggulsterone in both cells. The results showed that z-guggulsterone dose-dependently induced SGC-7901 apoptosis (Figure 2(a)), while GES-1 cells were only minimally affected by incubation with 75 *µ*M z-guggulsterone (Figure 2(b)).

### 3.4. Z-Guggulsterone Induces Apoptosis through the Mitochondrial Apoptotic Pathway in SGC-7901 Cells

**Figure 3:**
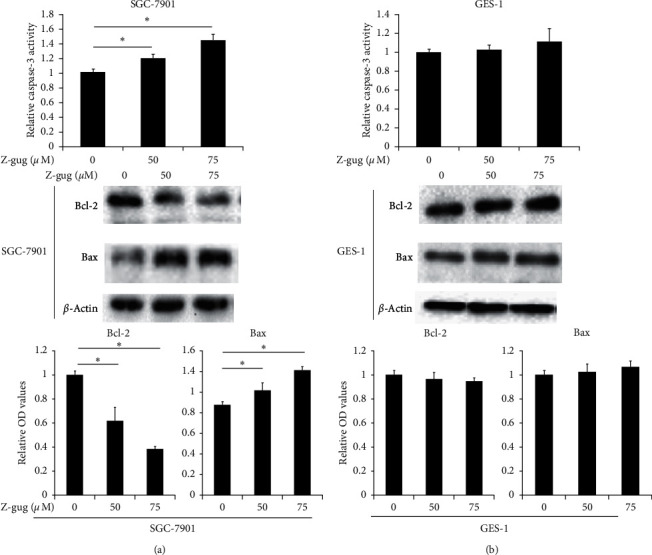
Z-guggulsterone affects the caspase-3 activity and the Bax and Bcl-2 expression in SGC-7901 cells. Cells were incubated with 0, 50, and 75 *µ*M z-guggulsterone for 24 h. (a) Caspase-3 activity level was assessed by ELISA. Data are expressed as means ± SD. ^*∗*^*p* < 0.05 vs. untreated cells. (b) Protein expression of Bcl-2 and Bax was evaluated by western blotting. Quantification of protein bands are shown in the bottom panels. *β*-Actin was used as a control. Data are expressed as means ± SD. ^*∗*^*p* < 0.05 vs. untreated cells. Z-gug, z-guggulsterone.

To determine whether mitochondrial-mediated intrinsic pathway was involved in z-guggulsterone-induced SGC-7901 cell apoptosis, expression levels of active caspase-3, Bax, and Bcl-2 were investigated. Z-guggulsterone dose-dependently increased the expression of active caspase-3 in SGC-7901 cells (Figure 3(a)). Bax expression was upregulated, but Bcl-2 expression was downregulated in SGC-7901 cells incubated with increasing concentration (0–75 *µ*M) of z-guggulsterone (Figure 3(b)). However, active caspase-3, Bax, or Bcl-2 expression was only minimally affected in GES-1 cells incubated with 75 *µ*M z-guggulsterone (Figures 3(a) and 3(b)).

### 3.5. Z-Guggulsterone Functions on the Cytokines TNF-*α*, VEGF, and TGF-*β*1 in SGC-7901 Cells

**Figure 4:**
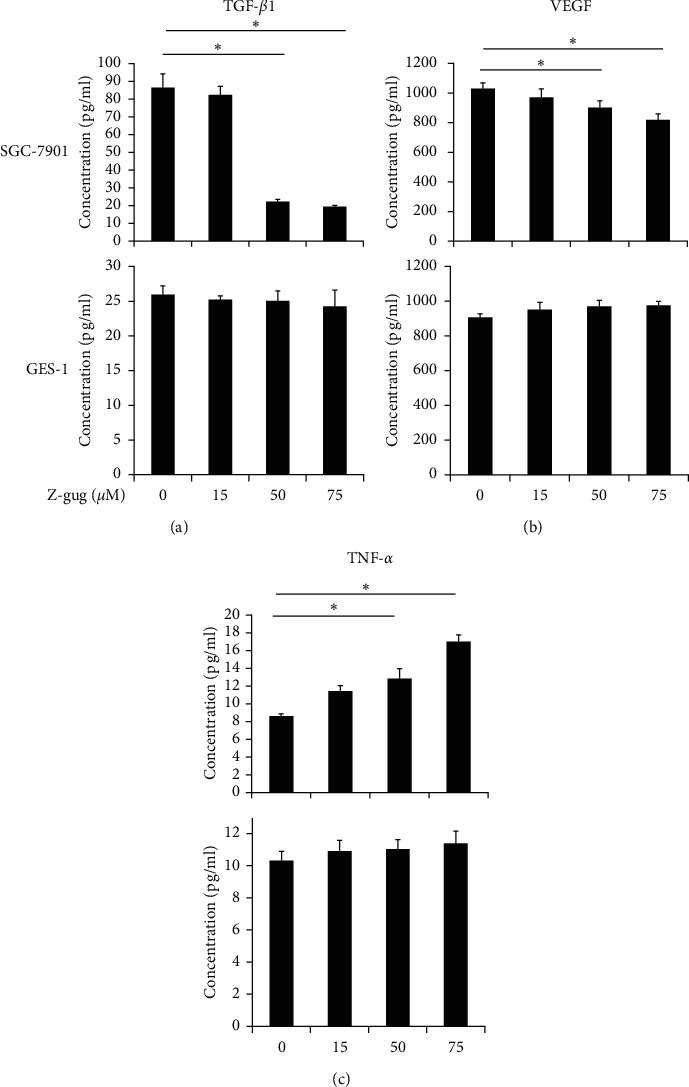
Z-guggulsterone alters TGF-*β* 1, VEGF, and TNF-*α* levels in SGC-7901 cells. SGC-7901 (a) and GES-1 (b) cells were treated with 0, 15, 50, or 75 *µ*M of z-guggulsterone for 24 h. TGF-*β*1, VEGF, and TNF-*α* levels in the culture medium were investigated using ELISA. Data are expressed as means ± SD. ^*∗*^*p* < 0.05 vs. untreated cells. Z-gug, z-guggulsterone.

When compared to untreated cells, VEGF and TGF-*β*1 levels were greatly decreased (*p* < 0.05), < while TNF-*α* content was greatly increased in SGC-7901 cells (*p* < 0.05) < following incubation with increasing concentration (0–75 *µ*M) of z-guggulsterone (Figure 4(a)). However, TNF-*α*, VEGF, and TGF-*β*1 contents were only minimally affected in GES-1 cells following incubation with 75 *µ*M z-guggulsterone (*p* > 0.05) (Figure 4(b)).

## 4. Discussion

Gastric cancer causes increasing cancer-related deaths throughout the world. The chemotherapy used to treat gastric cancer kills not only cancer cells but also normal cells by inducing toxicity and apoptosis. As a promising herbal medicinal agent, z-guggulsterone has been investigated thoroughly to cure numerous malignancies including gastric cancer [4]. Z-guggulsterone has been investigated to function as an antagonist of FXR to inhibit cell survival and induce cancer cell death [6, 9]. However, the role of z-guggulsterone in the cell apoptosis of gastric cancer is not completely clear. Our results indicated that the expression of FXR and its downstream target SHP is higher in SGC-7901 cancer cells compared with GES-1 normal gastric cells. Z-guggulsterone greatly attenuated the high expression of FXR and SHP. Meanwhile, the proliferation of SGC-7901 cells was greatly suppressed by z-guggulsterone dose-dependently. However, compared with SGC-7901 cells, gastric epithelial cells GES-1 were minimally affected by z-guggulsterone.

There is growing evidence that z-guggulsterone was involved in the mitochondrial apoptotic signaling pathway to prevent cancer growth [9, 15, 16]. We also found that z-guggulsterone dose-dependently induced apoptosis of SGC-7901 cells. Our results also indicated that active caspase-3 and proapoptotic protein Bax expression was increased, while antiapoptotic protein Bcl-2 expression was decreased in SGC-7901 cells under treatment of z-guggulsterone, indicating z-guggulsterone has a proapoptosis effect through the intrinsic mitochondrial pathway [17]. The cytokines TGF-*β* and TNF-*α* regulate apoptosis in various cancer cells and are associated with carcinogenesis [18]. VEGF is a characteristic factor for angiogenesis and cell viability during cancer progression [19]. Our results indicated that z-guggulsterone dose-dependently raised the level of TNF-*α* and reduced the levels of TGF-*β*1 and VEGF in gastric cancer cells. In the future, we would like to make clear the underlying mechanism for z-guggulsterone-induced TNF-*α* increase as well as TGF-*β*1 and VEGF decrease, which might be through their transmembrane receptors [5, 8].

The accumulated data have shown that the potential anticancer activity of z-guggulsterone is related to the induction of apoptosis. More specifically, z-guggulsterone altered the Bcl-2 family proteins and regulates FXR, NF-*κ*B signaling, MAPK signaling, EGFR, and Jak2-STAT3 signaling pathways in various cancer cells [15, 16, 20]. However, the mechanism underlying z-guggulsterone anticancer effects in gastric cancer cells is not completely understood. Here, we showed that z-guggulsterone exerted cytotoxicity on gastric cancer cells through inhibition of FXR signaling. Moreover, the mitochondrial intrinsic apoptosis pathway also participated in the chemotherapy process of z-guggulsterone in gastric cancer.

We also found that the normal gastric cell GES-1 was resistant to z-guggulsterone-induced cell death. Z-guggulsterone treatment did not significantly inhibit cell growth or promote apoptosis in GES-1 cells. There were no changes in mitochondrial-related active caspase-3, Bcl-2, and Bax protein expression, or in TNF-*α*, VEGF, and TGF-*β*1 level, in GES-1 cells with z-guggulsterone treatment. Thus, our findings showed that when compared with gastric cancer cells, normal gastric mucosa cells were less affected by z-guggulsterone, similar to results obtained in colon cancer [9], liver cancer [10], and others.

## 5. Conclusions

We demonstrated that FXR signaling antagonist z-guggulsterone reduced gastric cancer cell viability through inhibiting cell proliferation, decreasing intrinsic mitochondrial apoptosis, and downregulating VEGF and TGF-*β*1 levels but upregulating the TNF-*α* level. Our findings demonstrated that z-guggulsterone exerted as a potential herbal medicinal agent against stomach neoplasms and functioned through decreasing cell survival and regulating the levels of important growth factors/cytokines.

## Data Availability

The data used to support the findings of this study are available from the corresponding author upon request.
